# Out of an Abundance of Caution: COVID-19 and Health Risk Frames in Canadian News Media

**DOI:** 10.1017/S0008423921000214

**Published:** 2021-02-18

**Authors:** Rebecca Wallace, Andrea Lawlor, Erin Tolley

**Affiliations:** 1 Department of Political Science, University of Toronto Mississauga, 3359 Mississauga Rd., Mississauga, ON L5L 1C6; 2 Department of Political Science, King's University College at Western University, 266 Epworth Ave., London, ON N6A 2M3; 3 Department of Political Science, Carleton University, 1125 Colonel By Drive, Ottawa, ON K1S 5B6

**Keywords:** COVID-19, risk, public policy compliance, narratives, health, COVID-19, risque, confirmité aux politiques publiques, récits, santé

## Abstract

Although Canada's first documented case of COVID-19 appeared in mid-January 2020, it was not until March that messaging about the need to contain the virus heightened. In this research note, we document the use of the media's construction of risk through framing in the early stages of the pandemic. We analyze three dimensions of the health risk narratives related to COVID-19 that dominated Canadians’ concerns about the virus. To capture these narratives, we examine print and online news coverage from two nationally distributed media sources. We assess these frames alongside epidemiological data and find there is a clear link between media coverage, epidemiological data and risk frames in the early stages of the pandemic. It appears that the media relied on health expertise and political sources to guide their coverage and was responsive to the public health data presented to Canadians.

## Résumé

Bien que le premier cas documenté de COVID-19 au Canada soit apparu à la mijanvier, ce n'est qu'en mars que les messages sur la nécessité de contenir le virus se sont intensifiés. Dans cet article, nous documentons l'utilisation des récits de risque à la lumière de la recherche qui démontre le comportement individuel et la conformité aux politiques publiques. Nous analysons trois dimensions - la gravité, l'autorité et la proximité - des récits sur les risques sanitaires liés à la COVID-19 qui ont dominé les inquiétudes des Canadiens au sujet du virus. Pour saisir ces récits, nous examinons la couverture de la presse imprimée et en ligne de deux sources médiatiques diffusées à l'échelle nationale. Nous évaluons ces exposés parallèlement aux données épidémiologiques et constatons qu'il existe un lien clair entre les données de déclaration des cas et l'utilisation des récits dans les médias. Nous observons que, par le biais des médias, les Canadiens ont pu connaître l'avis de professionnels de la santé et d'autorités publiques faisant contrepoids aux considérations de risque. Nous suggérons que les récits non alarmistes en regard de la propagation réelle du virus peuvent avoir facilité la conformité aux directives de santé publique.

When Canada's first case of COVID-19 was documented in mid-January, public health officials assured the public that the risk to Canadians was very low. As additional cases appeared, however, the risk narrative began to change. Provinces and territories declared states of emergency, as did some cities, and the federal government passed a suite of emergency measures. As these measures were being introduced, more than half of Canadians (55%) thought it was likely they or someone they know would contract the virus; an equal percentage feared there not being enough medical equipment (Abacus Data, 2020). As the pandemic unfolded, experts and elites helped to reshape these beliefs, as well as the resulting behaviour, through the risk assessments they constructed.

Health advice evolved from self-monitoring when returning from abroad, to hygiene measures and physical distancing, to the closure of borders and nonessential services. For the most part, compliance with health directives—including limiting social contact, avoiding nonessential travel and working from home—was relatively high (Merkley et al., 2020, supplementary materials). Individuals seemed to understand the personal advantage of avoiding infection and the penalties for noncompliance, as well as the collective advantage of preserving healthcare resources by reducing transmission rates.

Existing research shows the media played a role by framing the pandemic primarily as a health crisis (Poirier et al., 2020). This research note extends this analysis by focusing on the media's construction of risk in the early stages of the pandemic, specifically in terms of framing the severity and proximity of the virus. We also consider the source of the risk framing, comparing the operational and strategic interventions offered by public health officials, on the one hand, and politicians and government authorities, on the other. By assessing these frames alongside epidemiological data, we conclude the media offered Canadians non-sensationalized coverage that corresponded quite closely with the virus's actual trajectory. The media's framing seems to have been principally informed by data and real-world events, rather than driven by hype or moral panic. Canadians received messages from the media, health professionals and public authorities that balanced considerations of risk, including the pandemic's severity and proximity, and this may have played a role in their overall compliance with public health directives. For political scientists, this finding is significant because it illustrates the interplay between the media, practitioners and political actors to encourage policy compliance and social consensus in a time of uncertainty.

## Media Framing, Risk and Public Policy Compliance

News media serve as the major conduit through which people learn about issues affecting their political communities, especially in disaster scenarios when individuals are struggling to understand the world around them (Crow and Lawlor, 2016; Lawlor and Crow, 2018). Amid health and environmental crises, “citizens tend to turn to the media for a greater understanding, but at the same time media and journalists are part of the process of framing science as certain or uncertain knowledge” (Jönsson, 2011: 123). In playing an active role in constructing our understandings of crises, news media highlight the various dimensions of risk associated with them.

Communication of risk is vital to public policy compliance because how individuals understand the nature, severity and proximity of risk influences their reaction to proposed interventions and, by extension, the efficacy of those interventions (Kiss et al., 2020). Existing research shows that citizens look to elites and experts to understand complex problems, and the information shared by these sources helps shape citizens’ views and behaviour (Druckman, 2001; Lachapelle et al., 2014). An individual's response is shaped by their underlying worldview and their impressions of the information source's credibility, but these moderators seem to matter less in high-risk policy domains or when an issue is new and emerging (Lachapelle et al., 2014; Zaller, 1992). Research conducted in the early months of 2020, as cases of COVID-19 were growing, suggests elites and the Canadian public were in a “rare moment” of cross-partisan consensus (Merkley et al., 2020). The current study demonstrates that consensus extended, in large part, to health officials and public authorities, as well as to the media, which framed the crisis in a manner that corresponded to its actual severity and proximity.

Most Canadians learned about the virus's trajectory and potential harm through the media. Although public reception to scientific information can vary and often depends on the degree of consensus within the scientific community—as well as individuals’ values, baseline science knowledge and trust in scientific researchers (Fischhoff and Scheufele, 2013; Hahn et al., 2016; Suhay and Druckman, 2015)—media frames about risk can shape public attitudes and behaviour. Such effects have been observed in support for water fluoridation (Perrella and Kiss, 2015), responses to climate change (Jones, 2014) and in the aftermath of disasters (Vasterman et al., 2005). However, if the media, public health officials and governments overplay their hand—if they exaggerate, overreact or sensationalize—this can dampen adherence to health directives (Anzur, 2000; Vasterman et al., 2005). For example, in the United States, right-leaning media disseminated false information and conspiracy theories about the virus, and consumers of those news sources were significantly more likely to endorse misinformation about the pandemic (Motta et al., 2020). Those narratives may have helped shape citizens’ views and behaviour, resulting in a different trajectory of case growth.

This research note looks at risk in three ways. First, we analyze news media frames related to COVID-19's severity and spread, documenting if and how the framing changed alongside epidemiological data. There is some evidence of the media sensationalizing health risks in other contexts (Vasterman et al., 2005), so our aim is to determine if the media's portrayal of risk is disproportionate to the case data in Canada.^1^ The media could have framed the virus as a distant foreign threat (risk attenuation) or an unstoppable foreign invader (risk amplification), but given evidence of limited polarization and general compliance with public policy directives, a more balanced narrative is possible. Consequently, we expect that as Canadian cases of COVID-19 increase, the framing around risk severity will intensify but these narratives will not deviate dramatically from the virus's actual trajectory.

Second, this analysis compares the source of health information, drawing on Boin and ’t Hart's (2010) theorization of the two levels of crisis response: (1) operational, which includes the medical personnel, epidemiologists and public health professionals focusing on the immediate threat, and (2) strategic, which includes political authorities who work in tandem with those in operations but whose role is more tactical and public-facing and who ultimately must account for the state's management of the crisis (see also Weible et al., 2020). We compare the number of risk frames offered by public health officials and epidemiologists with those coming from political sources, including the prime minister, premiers and mayors. As Canadian cases of COVID-19 increase, we expect operational experts to become more central in news coverage and for coverage to increasingly rely on the technical expertise offered by public health authorities, as opposed to messaging from political sources.^2^

Finally, this research note turns to frames on the proximity of risk, in order to assess whether COVID-19 was presented as a generalized or distant risk versus a more personal or proximate risk—and if and how this changed over time. There are many ways to interpret proximity of risk as it relates to the pandemic, such as measures of personal versus communal experiences with the virus or safety and hygiene practices. However, given that this analysis focused on the initial wave of the pandemic, proximity is conceptualized as physical vicinity or exposure to cases; this allows us to track whether the frames shifted as cases moved from China and Europe to North America and to the different provinces and cities within Canada. Although COVID-19 continues to affect the global community, the risk frames are likely to become more domestic, rather than international, as the number of new COVID-19 cases in Canada increases.

Our analytical focus builds on recent research on COVID-19 risk perceptions. For example, Dryhurst et al. (2020) highlight the cognitive and temporal-spatial dimensions of COVID-19's transmission as key aspects of risk perception, which include concerns about contracting the virus, its spread within one's country of residence and its perceived seriousness or impact on one's health. Perceived seriousness and contraction are captured in our measure of severity, which includes terms and phrases associated with transmission. We also assess severity by comparing the media's coverage of COVID-19's effect on health outcomes alongside the coverage of recovery and containment. Similarly, our interpretation of proximity assesses whether COVID-19 was communicated as a problem affecting countries outside Canada (“over there”) or within Canada (“at home”). Our over-time analysis allows us to test whether news framing of risk shifted to capture concerns associated with various sites of contraction, such as international travel versus community-based contraction, as the virus evolved. By analyzing the trajectory of these frames, we document how the media covered the virus and measure whether media framing of risk was consistent with epidemiological markers of its evolution.

## Data and Method

To understand the risk frames, this analysis draws on mainstream print and online news coverage from two main sources—Postmedia and the Canadian Press—which both have widespread national and local reach. These news organizations were selected because they represent many of Canada's top national, regional and city news outlets, including the CBC, CTV News, the *Globe and Mail*, *National Post* and others.^3^ Most factual information about COVID-19 came from elites and experts who used mainstream media as a conduit to the public.^4^ Although social media played a role in information dissemination, often the information in those sources reproduced that which was captured by the traditional media, and certainly from press briefings. Media coverage was thus a crucial source of information, particularly after lockdown measures limited interpersonal communication.

The media sample includes every news story published between March 1 and May 31 that mentioned COVID-19 (or coronavirus) in the headline or lead paragraph (*n* = 10,235 articles).^5^ We adopt a dialogical approach to automated analysis to uncover how specific dimensions of risk—including severity, proximity and source—are communicated in coverage of the pandemic.

Using computer-assisted content analysis, we developed keyword dictionaries to identify and measure the frequency of each risk frame (see online appendix A for details on this process). These dictionaries identified risk frames according to their Severity (transmission versus recovery), the Authority of the information source (public health versus political), and the Proximity of the risk (domestic versus international). In building the dictionaries, we adopted both deductive and inductive processes for identifying keywords and codifying news texts (see Table 1 for illustrative examples). For example, the research team—in addition to examining the most frequent terms and phrases in the text to construct the foundation of our dictionaries—read a large sample of articles to learn about the natural or common phrasing and terminology that journalists use in communicating about COVID-19 and the contexts in which these terms or phrases emerge, in order to expand our dictionaries. This approach ensured that our dictionaries were based on the language employed in the sample and captured risk as it was written into coverage. We then conducted a series of manual checks (detailed in online appendix A) to ensure that the dictionaries were valid. We match the news media database with epidemiological data on the number of new COVID-19 cases per day.^6^ We present a descriptive analysis of risk frames in the media alongside data on the actual spread of the virus.

**Table 1:** Frames with Illustrative Quotes

Frame		Keyword examples	Sample story quotation
Severity	*Transmission*	spread, death, ICU	“Health officials in Ontario said Friday that between 3,000 and 15,000 people could die of COVID-19 in the province even with the public health measures that are in place. Their projections suggested the death toll might have climbed to 100,000 without strong public health measures.” (Canadian Press, April 3, 2020)
	*Recovery*	reopen, resolve, slow the spread	“The decision was announced by Premier Jason Kenney on Wednesday, just hours before businesses were expecting to reopen. He said they are moving forward with plans to ease restrictions for the remainder of Alberta, which will see certain places, like restaurants, retail shops, hair salons, daycares and some dental and medical services resume operations on Thursday.” (Postmedia, May 14, 2020)
Authority	*Health authorities*	medical officer, epidemiologist, public health authority	“The province's chief medical officer encouraged parents not to take their kids to enclosed public spaces such as museums and shopping malls.” (Canadian Press, March 14, 2020)
	*Political authorities*	prime minister, premier, mayor	“Deputy Prime Minister Chrystia Freeland called on Canadians to be patient as the government worked to enact the unprecedented new measures, saying Ottawa is departing from the norm by focusing on speed rather than perfection.” (Canadian Press, March 21, 2020)
Proximity	*Domestic*	nursing home, school, restaurant	“The largest non-profit nursing home in Nova Scotia is reporting two more deaths from COVID-19, bringing the total to five in two days, amidst what the premier described as ‘a devastating day’ in the province.” (Canadian Press, April 19, 2020)
	*International*	travel, airport, cruise, overseas	“All of the cases have been linked to recent travel or have been in close contacts—such as spouses—of those infected while travelling.” (Canadian Press, March 10, 2020)

*Note*: See the online appendix for a complete list of keywords, Boolean operators, relevant stop and exclusion words, as well as information on the construction of the dictionaries.

## Results

**Figure 1:**
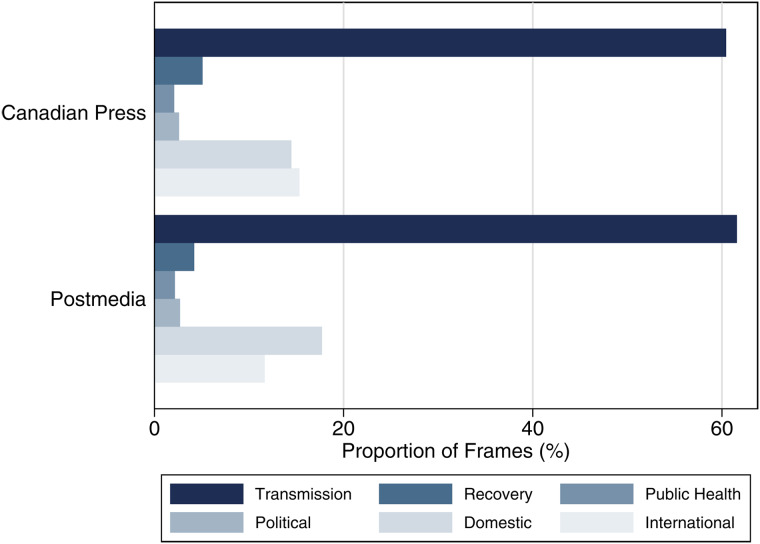
Proportion of Frames by Source

Figure 1 displays the proportion of risk frames presented in the news media database from the beginning of March to the end of May. During this time, an overwhelming proportion of frames focuses on the transmission of the virus. Despite the daily press briefings by political and public health figures, Authority frames were the least present in the data. We neither expect nor see an overwhelming difference in frame use by media source. Despite the slight ideological lean to the right of Postmedia, both media sources followed the same frame structure, with few exceptions. For example, Postmedia sources were more likely to use Domestic Proximity frames than the Canadian Press (*t*-test difference significant at the .001 level), while the Canadian Press sources were more likely to employ International Proximity frames (also significant at the .001 level).

**Figure 2:**
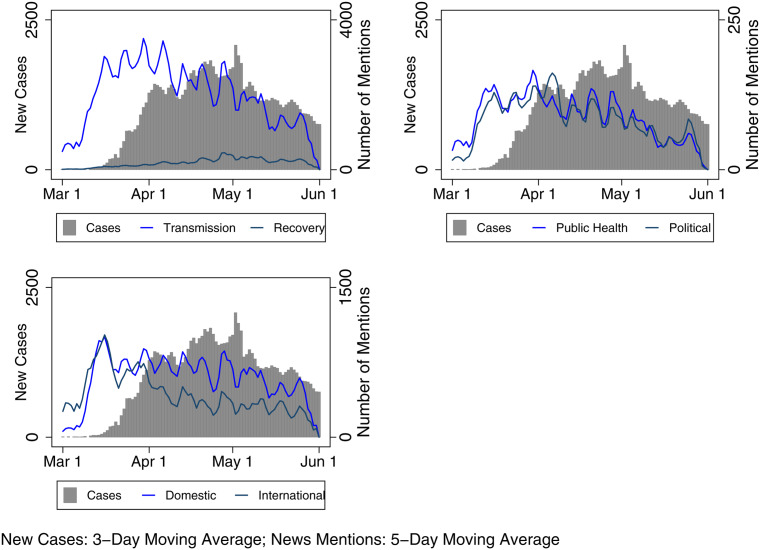
Media Frame Use (5-Day Moving Average) Overlaid with New Case Data (3-Day Moving Average)

Figure 2 shows the evolution of the frames and overlays Canada's COVID-19 case data to identify the relationship between media frames and new cases of the virus. The figure presents a three-day moving average of the number of new cases to smooth periods (for example, weekends) where testing may have been more limited, and we use a five-day moving average (current day plus four future days) in our counts of frame terms to account for the lag in news media adapting to the evolving number of cases. There are some clear associations between case data and frame use.^7^ The first panel of Figure 2 shows the contrast between Transmission and Recovery frames. The frequency of the Transmission frame increased sharply in relation to closure announcements from March 13 to 15 and is followed by a slow trend downward, which starts to mirror the trends in epidemiological data around mid-April. Interestingly, there is no corresponding increase around the surge in cases in early May, though this may be due to the familiarity with the pandemic by that point and the fact that many major aspects of coverage, such as the rollout of the Canada Emergency Response Benefit, had already occurred. By contrast, the Recovery frame does not emerge beyond negligible levels until the end of April and beginning of May (coinciding with a slow reopening in some provinces) and does not break through the frequency of the Transmission frame until the end of May. This pattern reflects the gradual rise in discussions about multiphased plans for reopening local economies. As Canadians became more familiar with the virus and how to limit its spread, the focus shifted to Recovery, though the risk posed by COVID-19 (and the Transmission frame) remained relatively high.

When we examine the authorities cited in news coverage (panel 2), we find little differentiation in the volume of references to health experts and political elites. This result is perhaps not unexpected because although the responsibilities and autonomy of medical officers of health vary across Canada (Fafard et al., 2018), it is likely there would be a greater degree of collaboration and consensus between health and political authorities during a public health crisis. At such times, there is a need to deliver clear and incisive guidelines on health measures to limit transmission rates, and decisions about communications—who says what—may be part of broader media and public relations strategies. There is just one notable exception in the data to this relative unity. Specifically, in advance of the Easter weekend, when there was concern that physical distancing protocols would be broken, we see a heightened frequency in the media's reliance on a Political Authority frame that advised individuals to remain sheltered in place with their immediate household. As one story noted, British Columbians were encouraged to “resist the urge to spend the Easter long weekend with loved ones outside their households” (Canadian Press, 2020). Interestingly, in the media's framing, there were no similar increases in the lead-up to Mother's Day and the Victoria Day long weekend, when similar concerns were publicly expressed.

Of greatest interest is the development of Domestic and International Proximity frames. After the mid-March closure of schools and nonessential businesses, the Domestic frame increases and overtakes the International frame, though both follow the same general trends. Here, increases in the number of new COVID-19 cases tend to produce a lagged increase in the use of Domestic risk terms. Although our focus is a descriptive record of the framing of the virus in the Canadian news media, we substantiate the connection between news frames and new cases in an analytical model available from the authors.^8^

**Figure 3:**
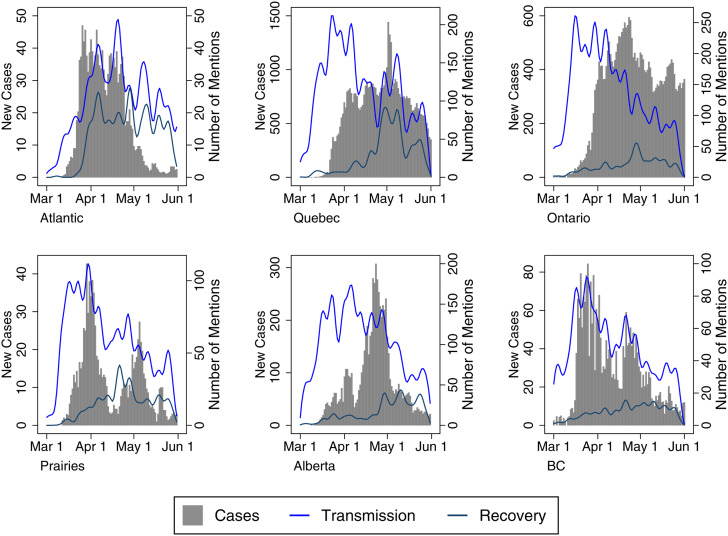
Severity Media Frames with Epidemiological Data (by Region)

Part of the COVID-19 story was a federal one, with a focus on international borders and the introduction of national assistance programs. Additionally, although delivery of health care is under provincial jurisdiction and decisions around testing, closures and the on-the-ground response had distinct regional angles, the federal government continued to play a critical role in public health, providing funding for applied research around the virus, for vaccinations and in support of provinces’ public health initiatives.^9^ Figures 3 through 5 explore the six frames using new case data from each region and news mentions that cued both the region and a frame term in the same sentence or paragraph (see regional dictionary in online appendix B). A manual verification of 5 per cent of dictionary mentions confirmed that these associations were capturing narratives that applied to each region (for example, “New Brunswick saw an increase in cases over the weekend.”)

The Transmission and Recovery frames in Figure 3 show some event-driven trends. First, Transmission frames increased when provinces declared states of emergency, and Transmission frame use peaks late for stories that mention the Atlantic provinces. Cases had already spiked in Atlantic provinces when media stories began to focus on regional transmission. Second, in British Columbia, a province lauded for its early response to the virus, the volume of Transmission narratives follows the case data almost exactly. However, although the province returned from lockdown earlier than most other provinces, there is not a particular surge in Recovery narratives. Third, in Alberta, Ontario and Quebec, the early wave of Transmission narratives seems to pre-date the swell of COVID-19 cases, which may have increased attentiveness to physical distancing guidelines, although we can only speculate. Finally, there are some localized peaks in the use of the Transmission frame. These are evident around the Cargill outbreak in Alberta in late April (Baum et al., 2020), large gatherings in a Toronto park in late May (Jones, 2020) and the alleged transmission of the virus by a New Brunswick doctor who travelled to Quebec in mid-to-late May (Humphreys, 2020). This coverage suggests the media were attentive to the risk of community transmission, a reality that is borne out in the case data.

**Figure 4:**
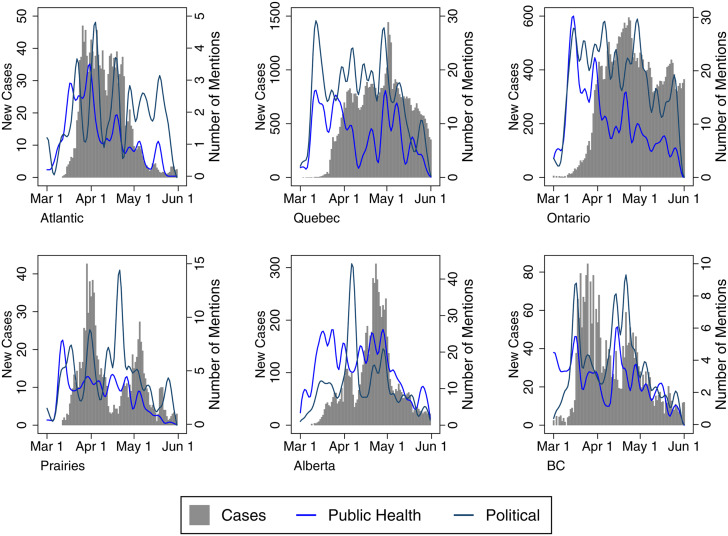
Authority Media Frames with Epidemiological Data (by Region)

Figure 4 illustrates the use of Health and Political Authority frames alongside case data by region. In some contexts, such as Atlantic Canada (in March), the Prairies (in March and May), Alberta (May to June) and British Columbia (mid-March onward), there is some degree of similarity in the frequency of Health and Political frames (with some exceptions where we see Political frames spike in response to local issues). Where the lines diverge, this is generally related to increased political messaging about reopening and economic recovery or coverage of key political announcements, including commentary by Alberta's premier on the withholding of medical supplies by the United States or the province's critical employment situation. The situation differed slightly in Ontario and Quebec, where there was a more sustained and substantial focus on provincial political authorities (though this gap narrowed in Quebec after May 1). In those jurisdictions, the health officials were less consistently lauded, and the relationship between them and their political counterparts became more fraught. For example, at the beginning of the outbreak, Quebec's public health director was mildly critiqued. He began to draw more favourable public commentary with his somewhat ostentatious online media presence, but this was disparaged as case numbers began to increase in the province. As officials mused about recovery, the public health director was portrayed as a “spin doctor” and lackey politicized by a government eager to reopen despite evidence of widespread community transmission (Freeman and Freeman, 2020; Macpherson, 2020). Meanwhile, throughout the pandemic, Ontario's chief medical officer has been roundly criticized for his poor public messaging with a communication style that is “confusing . . . befuddle[d] . . . impenetrable” (Arthur, 2020; Salutin, 2020). The government has emphasized repeatedly that it is taking advice from health experts, but the premier and key cabinet ministers have tended to carry a heavier load in press briefings. By contrast, Canada's chief public health officer, Dr. Theresa Tam, as well as the lead public health officials for British Columbia (the provincial health officer, Dr. Bonnie Henry) and Alberta (the chief medical officer of health, Dr. Deena Hinshaw) played more forward roles in the media commentary in the early stage of the pandemic.

**Figure 5:**
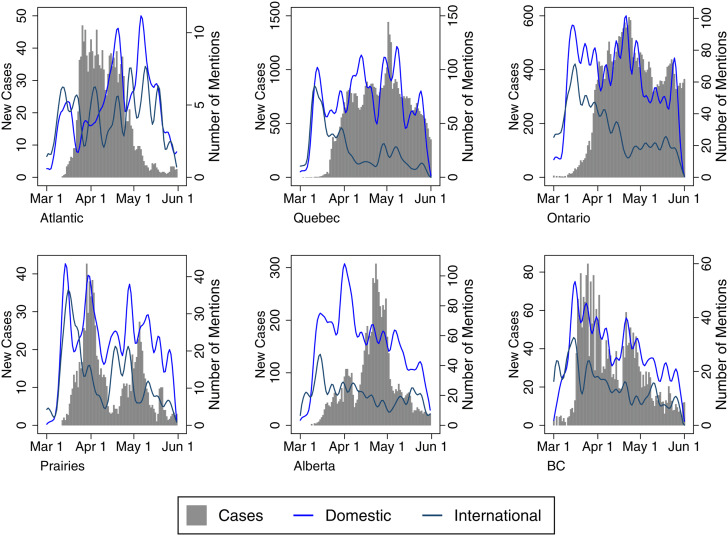
Proximity Media Frames with Epidemiological Data (by Region)

Finally, Figure 5 depicts the use of Domestic versus International frames to determine whether news coverage shifted alongside changes in the temporal-spatial risk perceptions over the timeframe under study. While coverage about the virus became far more (or almost all) domestic-centred in the months following our analysis, it is important to remember that the early stages of the pandemic were marked by a transition from hearing about the virus as a problem existing in other countries to one that reflected a growing number of cases domestically. Here, one might expect less regional variation in these risk frames, and there is an understandable emphasis on COVID-19 as a domestic issue in all regions. The Domestic frame follows case trends fairly closely in Quebec and Ontario; it also drastically outpaces international narratives in Quebec, Ontario, Alberta and British Columbia. Only in the Atlantic provinces do International frames outpace Domestic ones, partly because of a more intense focus on border restrictions in that region. For example, as a Postmedia article noted: “Nova Scotia declared its state of emergency on March 22, but immediately moved to ‘tighten’ its borders. All land, sea and air entry points are ‘closely managed’” (Humphreys, 2020).

## Frames alongside Evidence: The Relationship between COVID-19 Coverage and Cases

Our results suggest a correspondence between media coverage, epidemiological data and risk frames in the early stages of the pandemic. The media relied on health expertise and political sources to guide their coverage and did not exaggerate the risk posed to Canadians. Other research shows a remarkably high degree of elite and public consensus about the pandemic's threat, as well as fairly high compliance with health directives and social distancing rules (Merkley et al., 2020). Given the media's role in disseminating information about the virus, risk framing may have helped shape Canadians’ views about the virus's severity and proximity and encouraged compliance with public policy measures. Regional data confirm risk frames were consistent with context-specific COVID-19 issues, events and challenges across the Canadian provinces, which suggests the media were following the case data and aligning the narrative to operational and strategic information provided by health and political authorities.

Future studies will undoubtedly continue the analysis beyond May of 2020, as rates of COVID-19 continued to rise and fall, and public health measures changed accordingly. Researchers in public health would be well served to evaluate if and how frames pertaining to health risks change, particularly as provinces oscillate between stages of intense lockdown and lifting restrictions. Similarly, as subsequent waves of the virus response take hold, it will be important to assess whether the trends observed at the pandemic's outset return or whether media provide a more sensationalized approach to attempt to influence behaviour. Previous research shows that, early on, both English- and French-language Canadian news media framed the pandemic as a health crisis, but later on, there was some divergence as the English-language coverage began to focus more on social impacts than their French-language counterparts (Poirier et al., 2020). We recommend that our preliminary analysis be expanded to include coverage in both languages and to explore regional responses to recovery and ongoing discussions about the pandemic's more lasting effects. Future studies ought to also consider frames pertaining to economic risk and, perhaps more telling, how these correspond or diverge from the framing of health risks. Analyzing these dimensions of risk and how they are communicated will shed light not only on media coverage and public attitudes but also on broader compliance with efforts to curtail the virus.

## Notes

1 In this analysis, we define *sensationalism* as the disproportionate framing of risk on severity relative to epidemiological data pertaining to cases in Canada. This definition echoes the long-standing interpretation of sensationalism as the overstatement of risk in media (see, for example, Wahlberg and Sjoberg, 2000). We focus in this research note on the relationship (and correspondence) between the communication of severity and proximity and the epidemiological data. Future analyses could explore additional aspects of sensationalism, such as tone or message subject.

2 We expect that this would especially be true in the early months of the pandemic as information pertaining to the virus—including its contraction, side effects and consequences on the health care system—were still relatively unknown at that time. Reliance on health officials for safety guidelines was likely to increase along with the initial surge in Canadian cases during the first months of the pandemic.

3 Several of the newspapers represented by Postmedia and the Canadian Press also have the highest rates of readership in Canada (Newspapers Canada, 2015) and represent Canada's different regions. The latter consideration is central to this study, given the centrality of provinces and cities in response to the crisis. The sample includes an array of larger and smaller newspapers whose content is produced by Postmedia and Canadian Press.

4 Alluding to the media's role in information dissemination, Quebec's director of public health at one point referred to reporters as his “merveilleux pigeons voyageurs,” or “wonderful carrier pigeons” (Hachey, 2020).

5 Parameters pertaining to region were also incorporated in the database search to ensure the sample exclusively contained articles about COVID-19 in Canada or its regions.

6 https://www.canada.ca/en/public-health/services/diseases/2019-novel-coronavirus-infection.html.

7 Note that the left-hand axis (number of new cases) is standardized across the three subfigures; however, the right-hand axis (number of media mentions) is not to facilitate a clear view of the movement of the trends across time. Regional graphs vary both axes in order to see movement in data in smaller regions that would not be possible on a standardized axis.

8 To substantiate these visual trends, we use a Poisson regression model (available from the authors) to determine whether an upward change in the number of cases produces an increase in the media's use of particular narratives. This simple model is only meant to determine whether there is a significant relationship between change in the number of cases (lagged) and the number of narrative terms associated with each of our six categories of risk (expressed as a count).

9 Thank you to one of the journal's anonymous reviewers for this observation.

## Supplementary material

For supplementary material accompanying this paper visit https://doi.org/10.1017/S0008423921000214. click here to view supplementary material
