# Supplementary material for: Out of an Abundance of Caution: COVID-19 and Health Risk Frames in Canadian News Media
Source: Can J Polit Sci. 2021 Feb 18:1–14. doi: 10.1017/S0008423921000214 (PMC8082132; doi:10.1017/S0008423921000214)
Supplement: Supplementary file 1 [file S0008423921000214sup001.docx]

**Appendix A. Parameters pertaining to the content analysis**

To analyze this large volume of text, we used WordStat, a computer-assisted automated text analysis program. To explore the various dimensions of risk, we developed six dictionaries that applied to the various parameters of the COVID-19 risk. We compiled these dictionaries by reading random samples of news articles and selecting terms that appeared to signal the six dimensions of risk. This allowed us to explore the terminology that journalists and their sources used in conveying risk. We then built on that list and utilized the Keyword-in-Context function of WordStat to assess how the terms we selected were utilized across the sample before determining their fit under the various dictionaries. WordStat also allows researchers to apply specific rules or parameters for capturing keywords in the text; this allowed us to include or exclude certain uses of the terms to ensure that the dictionaries are capturing the appropriate use of such terms. For example, we when analyzing the use of the word “case” in context, most mentions referred to active or current cases; however, the word “recovered” sometimes preceded case, which helped to signify the mitigation of risk. As such, we included rules in our health risk dictionaries to include uses of the term “case” under high risk with certain exclusions to ensure it captured active or ongoing cases, and similarly included mentions of only recovered or resolved cases under low risk.

Although automated coding is generally very effective, especially considering the mass volume of text that can be analyzed in a timely manner, it is still important to manually assess that the results are valid. As such, we checked each dictionary term using the Keyword-in-Context function and looked at 20% of paragraphs where those terms were mentioned to ensure that the context, interpretation, and meaning were correct. Similarly, following from common practices in the field of automated content analysis (ex. Lawlor and Tolley 2017; Wallace 2018), we analyzed a random sample of 5% of the articles the corpus and conducted a manual check to ensure that the coding of the terms in our dictionaries were correct. Errors were corrected and capture-specific rules applied throughout this iterative process until there was at least an 85% agreement between automated and manual coding counts. In effect, this ensured that our data are reflective and captured what we believed they would.

The frames and sources explored in this analysis were also not mutually exclusive. The results of the analysis capture the references to dictionary terms throughout each article and, indeed, there are articles which refer, for example, to both political and public health authorities. The results reflect the total frequency of the dictionary terms in each article of the sample, and we generally refer to proportions of total references in the associated tables and figures for ease of comparison across the data more generally.

**Appendix B. Sample Dictionaries**

*indicate word stem / [text in square brackets indicate application of rules to data collection]

**Table B1. Categorization Dictionaries**

| **Transmission**  case* [case* not near resolv* not near recover*]  restriction* [restriction* not near lift* not near loos* not near eas*]  spread* [spread* not near stop_the]  critical_care  danger  death*  died  dies  distanc*  dying  emergenc*  first_wave  hospital*  ICU*  infect*  intensive_care  isolat*  kill*  next_wave  outbreak*  protect*  quarant*  respirator*  risk*  second_wave  symptom*  test*  ventilator* | **Recovery**  ease* [ease* near restrict* near guideline* near measure* near order*]  easing [easing near restriction* near guideline* near measure* near order*]  lift* [lift* near restriction* near guideline* near measure* near order*]  loose* [loose* near restrict* near guideline* near measure* near order*]  return* [return* near normal]  slow* [slow* near transmission* near progress* near spread* near risk*]  back_to_normal  flattened^[[1]](#footnote-1)^  mitigat*  re-open*  recover*  reopen*  resolve*  slow_the_spread*  under_control |
| --- | --- |
| **Health Authorities**  Barbara_Yaffe  Bonnie_Henry  Brendan_Hanley  Brent_Roussin  Deena_Hinshaw  Eileen_De_Villa  epidemiolog*  Heather_Morrison  Horacio_Arruda  infectious_disease_expert  infectious_disease_specialist  Janice_Fitzgerald  Jennifer_Russell  Kami_Kandola  medical_officer  Michael_Patterson  Mylène_Drouin  public_health_authorit*  public_health_officer  Robert_Strang  Saqib_Shahab  School of Public Health  Theresa_Tam  Patricia_Daly  Vera_Etches  virolog* | **Political Authorities**  Adrian_Dix  Assembly_of_First_Nations  Band_Council  Blaine_Higgs  Brian_Pallister  Cameron_Friesen  Caroline_Cochrane  Christine_Elliott  Clément_Chartier  Congress_of_Aboriginal_Peoples  Danielle_Mccann  David_Chartrand  Dennis_King  Diane_Thom  Doug_Ford  Dwight_Ball  François_Legault  George_Hickes  James_Aylward  Jason_Kenney  Jim_Reiter  Joe_Savikataaq  John_Haggie  John_Horgan  John_Tory  Justin_Trudeau  Kennedy_Stewart  Kim_Beaudin  Member_of_Legislative_Assembly  Member_of_Parliament  Member_of_Provincial_Parliament  Member_of_the_National_Assembly  MLA / MNA / MP / MPP  Métis_National_Council  Natan_Obed  Patty_Hajdu  Pauline_Frost  Perry_Bellegarde  Randy_Delorey  Robert_Bertrand  Sandy_Silver  Stephen_Mcneil  Ted_Flemming  Tyler_Shandro  Valerie_Plante  Scott_Moe |
| **Domestic**  care_centre*  care_facilit*  care_home*  community_transmi*  community_spread*  correctional_facilit*  daycare*  entertainment_facilit*  fitness_cent*  fitness_facilit*  gathering*  homeless*  librar*  long_term_care  museum*  nursing_home*  park*  recreation*  residence*  restaurant*  retirement_home*  school*  seniors'_home*  shopping_centre*  store*  seniors'_hous*  workplace*  transit | **International**  across_the_globe  airport*  around_the_globe  border*  China  countries  cruise*  Europe*  flight*  foreign*  global*  international  overseas  returned_from  returned_to_Canada  returning_from  travel*  trip*  U.S.  United_States  vacation* |

**Regional Dictionaries**

The regional dictionaries utilized in the study were intended to assess the extent to which the risk narratives were used in reference to different cities, counties/regions, and provinces/territories. How were the various dimensions of risk differently conceptualized or applied to the different regions within Canada? Although it is possible to derive some location data for news stories as a search parameter in the Factiva database, we opted to analyze region based on keyword mentions throughout the articles under study. We did so because many articles are comparative in nature, exploring how transmission and recovery rates – as well as different types of risks – affected different provinces, territories, and regions in Canada in different ways. As such, we built regional dictionaries that capture keyword references to the major cities, counties/townships/municipalities, regional health units, and provinces that make up the six regions under study in this analysis: British Columbia, Alberta, Prairies, Ontario, Quebec, and Atlantic Canada. The keywords in each dictionary were then used to created codes from which we could specifically analyze only sentences and paragraphs containing the regional cues with the other six risk dictionaries under study. This meant, for example, that we could exclusively analyze all paragraphs that included terms pertaining to Atlantic Canada (such as Newfoundland, PEI, Halifax, Saint John, etc.) and assess whether certain aspects of risk (such as domestic vs international risks) were more or less prominent in these paragraphs than paragraphs pertaining to the Prairies. Below are the six regional dictionaries included in the analysis.

**Table B2. Regional Dictionaries**

| **Alberta** |  |
| --- | --- |
| Alberta | Includes Alberta Health Services and Alberta Health |
| Calgary |  |
| Edmonton |  |
| Red Deer |  |
| Strathcona County |  |
| Lethbridge |  |
| Wood Buffalo |  |
| St. Albert |  |
| Medicine Hat |  |
| Grande Prairie |  |
| Airdrie |  |
| Stettler |  |
| Yellowhead |  |
|  | |
| **British Columbia** |  |
| British Columbia |  |
| BC | Includes BC Centre for Disease Control, BC Ministry of Health |
| Fraser Health |  |
| Interior Health |  |
| Northern Health |  |
| Vancouver | Includes Vancouver Coastal Health, Vancouver Island Health Authority, North Vancouver |
| Provincial Health Services Authority |  |
| Interim First Nations Health Authority |  |
| Surrey |  |
| Burnaby |  |
| Richmond |  |
| Abbotsford |  |
| Coquitlam | Includes Port Coquitlam |
| Kelowna |  |
| Langley |  |
| Saanich |  |
| Delta |  |
| Nanaimo |  |
| Kamloops |  |
| Victoria |  |
| Chilliwack |  |
| Maple Ridge |  |
| Prince George |  |
| New Westminster |  |
| North Vancouver |  |
|  |  |
| **Prairies** |  |
| Manitoba | Includes Manitoba Health |
| Interlake-Eastern Regional Health Authority |  |
| Northern Regional Health Authority |  |
| Prairie Mountain Health |  |
| Southern Health |  |
| Winnipeg | Includes Winnipeg Regional Health Authority, Winnipeg Health Region |
| Churchill Health Centre |  |
| Saskatchewan | Includes Saskatchewan Health |
| Athabasca Health Authority |  |
| Cypress Health Region |  |
| Five Hills Health Region |  |
| Heartland Health Region |  |
| Kelsey Trail Health Region |  |
| Keewatin Yatthé Regional Health Authority |  |
| Mamawetan Churchill River Health Region |  |
| Prairie North Health Region |  |
| Prince Albert Parkland Health Region |  |
| Regina | Includes Regina Qu'Appelle Health Region |
| Sun Country Health Region |  |
| Sunrise Health Region |  |
| Northern Inter-Tribal Health Authority |  |
| Saskatoon | Includes Saskatoon Health Region |
|  | |
| **Atlantic Canada** |  |
| New Brunswick | Includes New Brunswick Health |
| Horizon Health Network |  |
| Vitalité Health Network |  |
| Moncton |  |
| Saint John |  |
| Fredericton |  |
| Newfoundland | Includes Newfoundland and Labrador Department of Health and Community Services |
| Labrador | Includes Labrador-Grenfell Health |
| NL | Includes Service NL |
| Central Health |  |
| Eastern Health |  |
| Western Health |  |
| St. John's |  |
| Nova Scotia | Includes Nova Scotia Agriculture, Nova Scotia Department of Health and Wellness, Nova Scotia Environment |
| Annapolis Valley Health |  |
| Cape Breton District Health Authority |  |
| Capital Health |  |
| Colchester East Hants Health Authority |  |
| Cumberland Health Authority |  |
| Guysborough Antigonish Strait Health Authority |  |
| Pictou County Health Authority |  |
| South Shore Health |  |
| South West Health |  |
| Halifax |  |
| Cape Breton |  |
| Prince Edward Island | Includes Prince Edward Island Department of Health and Wellness |
| P.E.I. |  |
| PEI |  |
| Charlottetown |  |
|  | |
| **Ontario** |  |
| Ontario | Includes Ontario Ministry of Health and Long-Term Care, Public Health Ontario, Eastern Ontario Health Unit |
| Algoma | Includes Algoma Public Health |
| Brant County | Includes Brant County Health Unit |
| Chatham-Kent | Includes Chatham-Kent Health Unit |
| Durham | Includes Durham Region, Durham Region Health Department |
| Elgin-St. Thomas Health Unit |  |
| Grey Bruce Health Unit |  |
| Haldimand-Norfolk Health Unit |  |
| Haliburton, Kawartha, Pine Ridge District Health Unit |  |
| Halton | Includes Halton Region Health Department, Halton Hills |
| Hastings & Prince Edward Counties Health Unit |  |
| Huron County | Includes Huron County Health Unit |
| Kingston | Includes Kingston, Frontenac and Lennox & Addington Public Health |
| Lambton | Includes Lambton Health Unit |
| Leeds, Grenville and Lanark District Health Unit |  |
| Middlesex-London Health Unit |  |
| Niagara | Includes Niagara Region Public Health, Niagara Falls |
| North Bay | Includes North Bay Parry Sound District Health Unit |
| Northwestern Health Unit |  |
| Ottawa Public Health |  |
| Oxford County | Includes Oxford County Public Health |
| Peel | Includes Peel Public Health, Peel Region |
| Perth | Includes Perth District Health Unit |
| Peterborough | Includes Peterborough County-City Health Unit |
| Porcupine Health Unit |  |
| Renfrew | Includes Renfrew County, Renfrew County and District Health Unit |
| Simcoe County |  |
| Sudbury | Includes Sudbury & District Health Unit, Greater Sudbury |
| Thunder Bay | Includes Thunder Bay District Health Unit |
| Timiskaming Health Unit |  |
| Toronto | Includes Toronto Public Health |
| Waterloo | Includes Region of Waterloo Public Health |
| Wellington | Includes Wellington-Dufferin-Guelph Public Health |
| Windsor | Includes Windsor-Essex County Health Unit |
| York Region | Includes York Region Public Health Services |
| Mississauga |  |
| Brampton |  |
| Muskoka | Includes Simcoe Muskoka District Health Unit |
| Mississauga |  |
| Brampton |  |
| Hamilton |  |
| London |  |
| Markham |  |
| Vaughan |  |
| Kitchener |  |
| Richmond Hill |  |
| Oakville |  |
| Burlington |  |
| Oshawa |  |
| Barrie |  |
| St. Catharines |  |
| Guelph |  |
| Cambridge |  |
| Whitby |  |
| Ajax |  |
| Milton |  |
| Brantford |  |
| Clarington |  |
| Pickering |  |
| Newmarket |  |
| Kawartha Lakes |  |
| Sault Ste. Marie |  |
| Sarnia |  |
| Caledon |  |
| Norfolk County |  |
| Aurora |  |
| Welland |  |
| Belleville |  |
|  | |
| **Quebec** |  |
| Quebec | Includes Institut national de santé publique du Québec, Quebec Ministry of Health and Social Services |
| Agence de la santé et des services sociaux de l'Abitibi-Témiscamingue |  |
| Agence de la santé et des services sociaux du Bas St-Laurent |  |
| Agence de la santé et des services sociaux de la Capitale-Nationale |  |
| Agence de la santé et des services sociaux de Chaudière-Appalaches |  |
| Agence de la santé et des services sociaux de la Côte-Nord |  |
| Agence de la santé et des services sociaux de l'Estrie |  |
| Agence de la santé et des services sociaux de la Gaspésie—Iles-de-la-Madeleine |  |
| Agence de la santé et des services sociaux des Laurentides |  |
| Agence de la santé et des services sociaux de Laval |  |
| Agence de la santé et des services sociaux de la Mauricie et du Centre-du-Québec |  |
| Agence de la santé et des services sociaux du Saguenay—Lac-St-Jean |  |
| Centre régional de santé et de services sociaux de la Baie-James |  |
| Nunavik Regional Board of Health and Social Services |  |
| Public Health Department of the Cree Health Board |  |
| Montreal | Includes Agence de la santé et des services sociaux de Montréal |
| Quebec City |  |
| Laval |  |
| Gatineau |  |
| Longueuil |  |
| Sherbrooke |  |
| Saguenay |  |
| Lévis |  |
| Trois-Rivières |  |
| Terrebonne |  |
| Saint-Jean-sur-Richelieu |  |
| Brossard |  |
| Repentigny |  |
| Drummondville |  |
| Saint-Jérôme |  |
| Granby |  |
| Blainville |  |
| Saint-Hyacinthe |  |
| Mirabel |  |
| Lanaudière | Includes Agence de la santé et des services sociaux de Lanaudière |
| Montérégie | Includes Agence de la santé et des services sociaux de la Montérégie |
| l'Outaouais | Includes Agence de la santé et des services sociaux de l'Outaouais |

1. Past tense only. “Flatten” was most often used in calls to “flatten the curve”, signifying that there was an imminent risk and need to act (although sometimes inconsistently, hence it was not used in the high risk dictionary); “flattened” was used to describe regions or cities where crisis was averted, signaling low risk. [↑](#footnote-ref-1)
